# Effects of Long-Term Administration of Gardeniae Fructus on Intra-Abdominal Organs of Rats

**DOI:** 10.1155/2020/4201508

**Published:** 2020-06-08

**Authors:** Hisato Takei, Seiichi Iizuka, Masahiro Yamamoto

**Affiliations:** ^1^Tsumura Kampo Museum, Corporate Communications Dept., TSUMURA & CO., Ibaraki 300-1192, Japan; ^2^Kampo Research and Development Division, TSUMURA & CO., Ibaraki 300-1192, Japan

## Abstract

Many recent reports have suggested a possible association between Japanese traditional (Kampo) medicines containing Gardeniae Fructus (GF, the fruit of *Gardenia jasminoides* J. Ellis) and the mesenteric phlebosclerosis (MP). MP is a chronic orphan disease characterized by venous calcification extending from the colonic wall to the mesentery, usually developing in the proximal colon. In the present study, we administered GF to Wistar/ST female rats as 1% and 2% feed in the diet for 11 months to evaluate any calcification and/or fibrosis of veins in the colonic wall and mesentery. The reversibility of GF's effects was examined by feeding a normal diet for an additional 3 months. A significant decrease in body weight gain and food consumption occurred in the 2% GF group. Pigmentation of the liver, kidney, and spleen in macroscopic or histopathological examination was observed after 11-month administration, which disappeared after the 3-month recovery period. Histopathological findings such as fibrous thickening and calcification of vein walls, characteristic of human MP, were not observed. Fibrosis in the colonic lamina propria was observed in the 2% GF group but not in the 1% GF group during the treatment period, but the incidence as well as grade of this type of fibrosis decreased in the recovery period, suggesting that the effects of GF were reversible. In the present study, chronic GF administration did not result in any venous pathological changes but induced pigmentation in the liver, kidneys, and spleen and moderate fibrosis in the colonic lamina propria, all of which being reversible. Further studies are required to determine the association between GF and MP.

## 1. Introduction

Mesenteric phlebosclerosis (MP) was first proposed by Iwashita and associates in 1993 [1]. It is defined as a chronic ischemic disease characterized by mesenteric vein sclerosis with unknown etiology. Reported findings in MP include the following histopathological changes of the intestinal wall: (1) fibrous thickening of the vein walls in the colon and calcification of blood vessel walls; (2) marked submucosal fibrosis and deposition of collagen around vessels in the mucosa; (3) absence of inflammatory cell infiltration; and (4) change in color of the colon to dark purple with a bronze tone [2–4]. Most cases of this disease have been reported from East Asia, especially Japan. In addition, MP has been reported in patients treated with Kampo medicine [5–16], particularly following long-term administration of Kampo formulas (e.g., Kamishoyosan) containing Gardeniae Fructus (GF) [6, 9, 14–16]. GF is the fruit of the plant *Gardenia jasminoides* J. Ellis. It contains geniposide, an iridoid glycoside, as the main constituent, which has been suggested as a probable cause of MP [17]. Geniposide is known to be converted to genipin by intestinal bacteria [18], and genipin produces pigments by chemically combining with amino bases [17]. In the present study, we investigated whether long-term administration of GF to rats could reproduce MP-like pathology.

## 2. Materials and Methods

### 2.1. Animals

Previous clinical studies suggest predominance of MP in females [7, 15, 16]; therefore, in the present study, female rats were used. Virgin female Wistar/ST rats (weight range, 70–90 g; age, 4 weeks upon receipt) were purchased from SLC (Shizuoka, Japan) and acclimated for 7 days. The animals were housed individually in plastic cages (315 mm *W* × 465 mm *D* × 200 mm H, Ishihara Co., Ltd., Tokyo, Japan) in an animal room under controlled conditions of temperature (23 ± 2°C), humidity (55 ± 10%), and lighting (12-h light/dark cycle). Ten animals were assigned to each group. Each animal was equipped with Laboratory Animal Tag (Scitec, Inc., Shizuoka, Japan). Animals were provided with food pellets (MF, Oriental Yeast Co., Ltd., Tokyo, Japan) and drinking water *ad libitum*. Treatment with GF was initiated when the animals were 5 weeks of age.

### 2.2. Drugs

A dried extract of GF (Lot No. 2121044020) was manufactured by Tsumura & Co. (Tokyo, Japan). The extract powder was mixed with the food pellets, which were given *ad libitum* for 11 months. The concentration of GF mixed into the feed was adjusted to 1.0% or 2.0%. The average exposure dose of GF in this study was approximately 459 mg/kg in the 1% GF group and 987 mg/kg in the 2% GF group. The doses in the present study are higher than the clinical doses (typically 0.125 g/kg body weight as GF extract). Because the focus of the present study is the investigation of possible harmful effects of long-term administration of GF, we first investigated the appropriate dose for long-term study of the effect of GF. Since acute oral administration of an aqueous GF solution at concentrations greater than 2.0% sometimes induced death in rats, the maximal concentration for the present study was determined to be 2.0%. Doses in this range are similar to those used in studies on the efficacy of GF and GF-containing Kampo medicines in animal models of liver disease [19, 20], in which GF exerted beneficial effects on liver function and pathology with no apparent toxicity.

### 2.3. Analysis of 3D HPLC Fingerprint of Gardeniae Fructus

**Figure 1:**
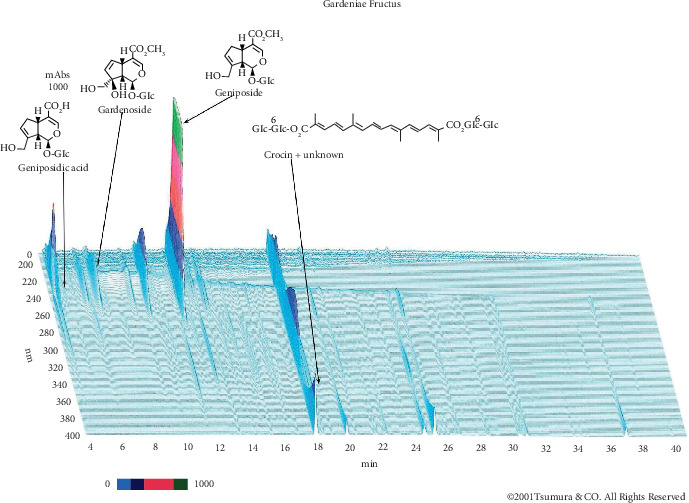
3D HPLC profile of Gardeniae Fructus (GF). Peak analysis and assignment were performed using standard samples that had been isolated from raw materials. The chromatographic conditions are described in the Materials and Methods section. Absorbance in mAbs; wavelength in nm; retention time in min.

GF (1.0 g) was extracted with methanol (30 mL) under ultrasonication for 30 min. The solution was filtered and then analyzed by HPLC. HPLC equipment was controlled with an HPLC pump (LC-10AD; Shimadzu, Kyoto, Japan) using a TSK-GEL ODS-80Ts column (4.6 mm i.d. × 250 mm), eluting with solvents (A) 0.05 M AcONH_4_-AcOH buffer (pH 3.6) and (B) CH_3_CN. A linear gradient of 90% A and 10% B changing over 60 min to 0% A and 100% B was used, and 100% B was continued for 20 min. The flow rate was controlled with an LC 10AD at 1.0 mL/min. The eluate from the column was monitored, and the three-dimensional data were processed by using a diode array detector (SPD-M10A_VP_; Shimadzu, Kyoto, Japan). A three-dimensional HPLC chart of the methanol solution of GF is shown in Figure 1.

### 2.4. Experimental Design

GF was administered orally to female rats at 0%, 1%, and 2% feed in the diet for 11 months (*n* = 20 for each group). At the end of GF treatment, 10 animals were sacrificed for histopathological examination. The remaining rats were maintained for 3 additional months without GF. Histopathological examination was performed at the end of the experiment.

### 2.5. Clinical Observations, Body Weight Gain, and Food Consumption

Animals were inspected at least once daily for evidence of treatment- or nontreatment-related signs or ill health during the first months from the recovery period onward. Body weight gain and food consumption were measured at least once a month throughout the 11-month administration period and recovery period in the treatment and nontreatment groups.

### 2.6. Macroscopic Observations

The rats were necropsied at the end of the GF administration period or the recovery period. At autopsy, all animals fasted overnight were anesthetized with isoflurane (Forane® inhalant liquid, Abbott Japan Co., Ltd., Tokyo, Japan) by inhalation. The surviving animals were euthanized on the next day after the end of administration. Gross observation was performed for the main organs and tissues containing the head, chest, and abdomen.

### 2.7. Histopathological Examination

After autopsy, tissues were fixed in 15% phosphate-buffered formalin, embedded in paraffin wax for routine processing, and sectioned at 3 *μ*m thickness. For light microscopy, sections of the ileum, colon, and mesentery were stained with hematoxylin and eosin (HE) and Masson trichrome (MT), and those of the spleen, liver, and kidneys were with HE i.e., rats that were necropsied at the end of the GF administration period (maximum 11 months); rats that were humanely killed in extremis during administration; rats that were necropsied at the end of the 3-month recovery period; and rats that were found dead during the recovery period. In addition, histopathological examination was performed on the HE-stained sections of the adrenal glands, stomach, duodenum, jejunum, cecum, rectum, pancreas, and macroscopic lesions of animals necropsied at the end of the 3-month recovery period, as well as the adrenal glands, stomach, duodenum, jejunum, cecum, rectum, pancreas, cerebrum, cerebellum, pituitary, eye, trachea, thyroid, parathyroid, heart, and lung of 1 animal that died during the recovery period. Histopathological examination was done at Tsukuba Institute, BoZo Research Center Inc. (Tokyo, Japan).

Individual histopathological findings for 30 rats that were treated for 11 months are shown in Table 1, and individual histopathological findings for 23 rats that recovered for 3 months are shown in Table 2, in accordance with the International Harmonization of Nomenclature and Diagnostic Criteria (INHAND) guide (https://www.goreni.org/).

### 2.8. Statistical Analysis

Statistical significance was determined using Dunnett's parametric multiple comparison test. A *p* value <0.05 was considered statistically significant. Data in figures represent mean ± SD.

### 2.9. Ethical Statement

This study was carried out in accordance with the recommendations in the Guide for the Care and Use of Laboratory Animals of the Japanese Association for Laboratory Animal Science. The protocol was approved by the Committee on the Ethics of Animal Experiments of Tsumura & Co. The experiments in the present study were designed to minimize the number of animals used.

## 3. Results

### 3.1. Clinical Signs, Body Weight Gain, and Food Consumption

Three animals receiving 2% GF died before day 233 of treatment. Two animals receiving 1% GF and 1 animal receiving 2% GF were killed in extremis on day 233 of treatment. Of the animals killed in extremis, signs prior to death included underactivity, abnormal gait, and ataxia due to body tumor or paralysis. In the recovery period, one 2% GF-treated animal was found dead on day 89 of the reversibility phase. Signs before death included marked emaciation, staggering gait, soiled fur, and underactivity. Signs of diarrhea were transiently observed in several rats in the 2% GF group. There were no other signs that could be equivocally associated with treatment.

**Figure 2:**
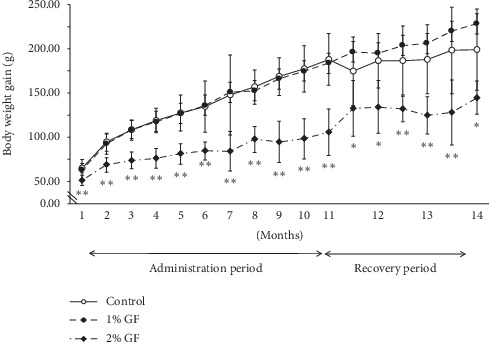
Body weight gain of rats treated orally with Gardeniae Fructus (GF) for 11 month followed by a 3-month recovery period. Vertical bars represent mean ± SD. ^*∗*^*p* < 0.05 vs. control; ^*∗∗*^*p* < 0.01 vs. control.

**Figure 3:**
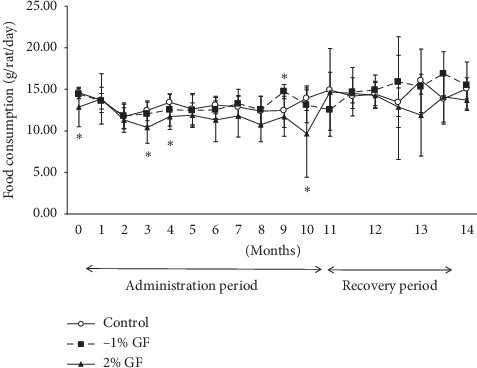
Food consumption of rats treated orally with Gardeniae Fructus (GF) for 11 month followed by a 3-month recovery period. Vertical bars represent mean ± SD. ^*∗*^*p* < 0.05 vs. control.

Decreased mean body weight gain was evident from the beginning of GF treatment at the 2% dose, while the decrease appeared to begin later in the 1% GF group (Figure 2). At several points during treatment, food consumption in the 2% GF treatment group was significantly lower than that in the control group (Figure 3). Food consumption during the 3-month recovery period showed no significant difference among the groups (Figure 3).

### 3.2. Macroscopic Findings

In the 1% GF group, discoloration of the liver (dark red), kidneys (dark green), and spleen (dark brown) was observed in 3/10, 9/10, and 6/10 rats, respectively. In the 2% GF group, discoloration (dark red or dark green), atrophy with diffuse decolorization in the liver was noted in 4/10, 4/10, and 2/10 rats, respectively. Discoloration of the kidneys (dark green) and spleen (dark brown) was observed in 10/10 and 8/10 rats, respectively.

Three rats in the control group showed subcutaneous polycysts. Rats in the 1% GF group showed subcutaneous polycysts (3 cases), dark greenish kidney (1 case), and subcutaneous tumor of the hypogastrium (1 case), while rats in the 2% GF showed subcutaneous polycysts (3 cases), white nest of the liver (2 cases), hematoma (1 case), dark greenish kidney (4 cases), dark greenish adrenal gland (1 case), and hypertrophy of the spleen (2 cases) during the 3-month recovery period. Other findings were considered incidental or spontaneous because they were observed in the control group at a similar rate of incidence and are known lesions associated with aging.

### 3.3. Histopathological Findings

**Figure 4:**
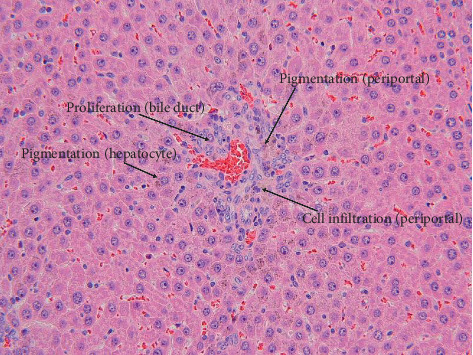
Liver from a rat at the end of the 2% GF treatment period. Bile duct proliferation: mild. Periportal lymphoid cell infiltration: mild. Periportal pigmentation (brown granular): mild. Hepatocyte pigmentation (brown granular): mild (H&E staining, ×40).

**Figure 5:**
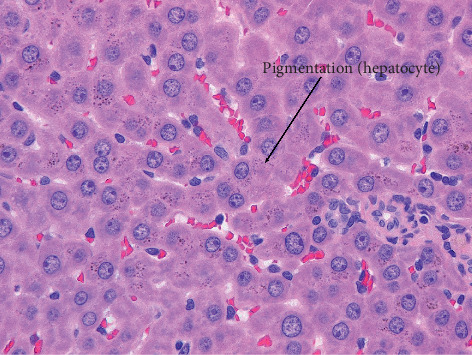
Liver from a rat at the end of the 2% GF treatment period. Hepatocyte pigmentation (brown granular): mild (H&E staining, ×40).

**Figure 6:**
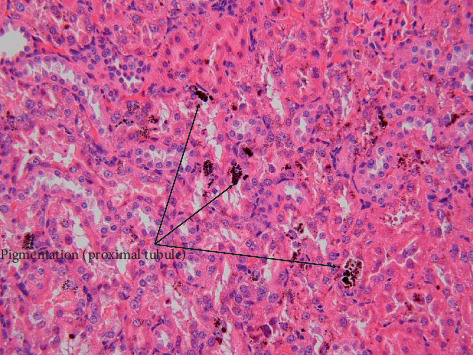
Kidney from a rat at the end of the 2% GF treatment period. Pigmentation in proximal tubule (brown granular): moderate (H&E staining, ×40).

The scores for histopathological findings in individual animals at the end of treatment period and recovery period are shown in Tables 1 and 2, respectively. Treatment-related histopathological changes were observed in the livers and kidneys of rats in the 2% GF group. The findings included intrahepatic bile duct proliferation and infiltration of lymphocytes, suggesting that GF is related to the degeneration of hepatocytes and regenerative proliferation of bile ducts. Although atrophy of hepatocytes and decreased vacuoles in periportal hepatocytes were observed (Figure 4), the hepatocyte results were considered to be due to the decrease in food intake rather than direct toxicity of GF [21, 22]. In addition, diffuse brown granular pigmentation was observed in hepatocytes (Figures 4 and 5) and renal tubular epithelial cells (Figure 6) in almost all the treated rats. No obvious histological changes, except for pigmentation or regeneration of hepatocytes, were observed in the examined organs of treated groups after the 3-month recovery period. Other findings were considered incidental or spontaneous because they were observed in the control group at a similar rate of incidence and are lesions known to be associated with aging.

**Figure 7:**
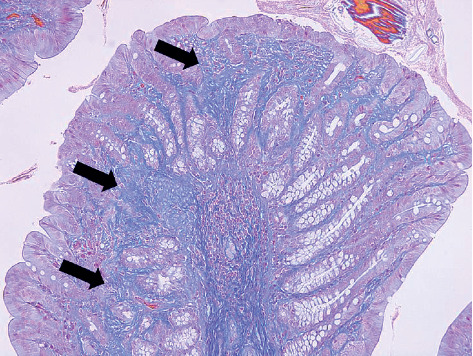
Colon from a rat at the end of the 2% GF treatment period. Lamina propria fibrosis: moderate (MT staining, ×20).

**Figure 8:**
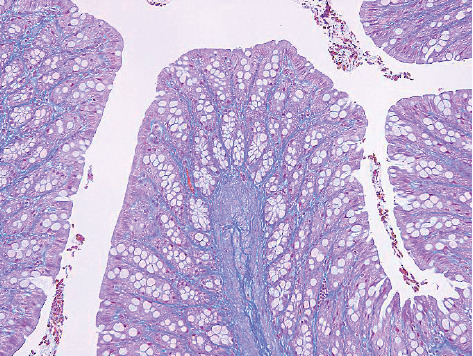
Colon from a rat at the end of the control treatment period. No remarkable aberration (MT staining, ×20).

**Figure 9:**
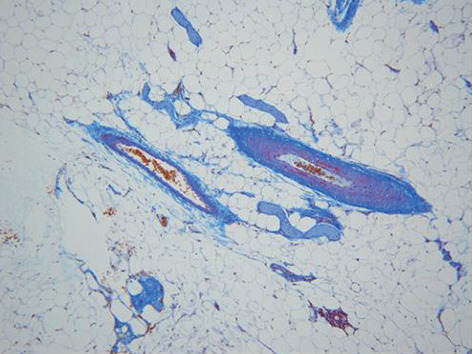
Mesentery from a rat at the end of the 2% GF treatment period. No remarkable fibrosis (MT staining, ×20).

In the colon, mild to moderate fibrosis in the lamina propria was observed in GF-treated rats (Figure 7), which disappeared after the 3-month recovery period. The fibrosis was not observed in the lamina propria of control sections (Figure 8). Treatment-related microscopic changes were not observed in the mesenteric vein (Figure 9).

Minimal inflammation in the lung bronchioli was considered to be related to malnutrition of this animal. Minimal focal accumulation of alveolar macrophages in the lung was considered incidental. No treatment-related findings were observed in other organs.

## 4. Discussion

The doses of GF used in the present study have been reported to be effective in various disease models with no apparent toxicity. However, it should be noted that a major GF ingredient, geniposide, has been shown to be acutely hepatotoxic at high doses [23, 24]. Possible toxicity of other GF ingredients has also been suggested [25]. In this study, pathological changes such as calcification and fibrosis of the mesenteric vein in the colon were not observed. Furthermore, purple discoloration of the colonic mucosa was also not observed. However, although the degree was very mild, fibrosis in the colonic lamina propria was observed, in addition to discoloration of the liver, kidney, and spleen.

Although chronic GF treatment did not reproduce human MP-like pathology in the present experimental setting, the possible involvement of GF in colonic discoloration associated with human MP cannot be ruled out. Geniposide is known to produce genipin by beta-D-glucosidase of intestinal bacteria [18]. Genipin reacts with amines, especially amino acids, and subsequently produces derivatives with various colors [17, 26, 27]. Pigmentation of the liver, kidney, and spleen in our macroscopic and histopathological examinations was also reported in a chronic toxicity study in SD rats, in which geniposide was administered orally at a dose of 25, 50, or 100 mg/kg/day for 26 weeks [17]. At a dose of 100 mg/kg/day, severe abnormalities and pigmentation were found in the liver and kidneys. Because generation of pigment from genipin proceeds slowly [27] and the turnover of intestinal mucosal cells is rapid, cumulative pigmentation due to chronic GF administration could be more observable in organs other than the intestines in small animals. It is unclear whether the liver and kidneys are discolored in human MP patients. Other GF ingredients, such as the hydrophilic carotenoid crocin, have also been reported to cause reversible black pigmentation in the liver at high doses [28, 29].

Aside from geniposide and crocin, GF contains various bioactive ingredients such as crocetin, geniposidic acid, and gardenoside [30]. Various pharmacological effects of crocin and crocetin, as well as toxicity, have been reported [31, 32]. Pharmacological activity has also been suggested for other GF ingredients, and in vitro studies suggest that many of them may have cytotoxic/hepatotoxic effects [25]. Therefore, investigation of GF ingredients and the mechanisms responsible for the pathogenesis of MP warrant further extensive studies.

In addition to the elucidation of the herbs and/or ingredients responsible for MP, it may be important in the future to clarify the effects of various combinations of ingredients and/or herbs. It is possible that a mixture of herbs decreases the adverse effect of toxic compound(s) contained in any specific herb. Several Kampo medicines contain GF. In addition to a detailed comparison of the clinical MP induced by different Kampo medicines, it would be also informative to determine whether the addition of other herbs changes the effect of GF, for example, discoloration and fibrosis in the colon of animals.

In the present study, chronic GF treatment increased collagen fiber and eosinophilic deposition in the lamina propria in 7/10 cases, although these changes were mild and not located around the periarterial/perivenous area of the mesentery. The incidence and grade of fibrosis decreased after the 3-month recovery period, suggesting that the fibrosis is reversible, which is consistent with clinical reports indicating that the symptoms of MP improve following cessation of administration of a Kampo formulation containing GF [7, 12, 14, 16]. It is noteworthy that, although it remains unclear as to whether discoloration is harmful, the discoloration of the liver, kidneys, and spleen in rats was also found to be reversible, as it is in the colon of MP patients. It remains to be clarified whether the fibrotic changes in the lamina propria induced by GF are related to MP.

## 5. Conclusion

In this study, we repeatedly administered GF for 11 months to determine whether chronic GF treatment reproduces human MP-like pathology in rats. Important characteristic symptoms of human MP, such as fibrous thickening, calcification of the colonic vein wall, and dark purple/bronze discoloration in the colon were not observed. Fibrosis in the colonic lamina propria was observed but the extent was very mild.

Furthermore, discoloration of the liver, kidney, and spleen was observed. All of the observed changes returned to the normal state 3 months after discontinuation of GF administration.

## Figures and Tables

**Table 1:** Individual histopathological findings in female rats during the 11-month treatment period.

Dose	Control										1% GF										2% GF									
Rat ID no.	01	02	03	04	05	06	07	08	09	10	11	12	13	14	15	17	18	19	20	21	31	32	33	34	35	36	37	38	39	41
Tissue observation																														
Ileum (H&E and Masson trichrome staining)	—	—	—	—	—	—	—	—	—	—	—	—	—	—	—	—	—	—	—	—	—	—	—	—	—	—	—	—	—	—
Colon																														
Increased height, villous	—	—	—	—	—	—	—	—	—	—	—	—	—	—	—	—	—	—	—	—	1	—	—	1	1	—	1	1	—	—
Deposit, eosinophilic, lamina propria	—	—	—	—	—	—	—	—	—	—	—	—	—	—	—	—	—	—	—	—	3	—	2	3	3	—	3	3	2	—
Cell infiltration, mesocolon, focal	—	—	—	—	—	—	—	—	—	—	—	—	—	—	—	—	—	—	—	—	—	—	—	—	—	—	—	—	—	—
Inflammation, mucosa, focal	—	—	—	—	—	—	—	—	—	—	—	—	—	—	—	—	—	—	—	—	1	—	—	—	—	—	—	—	—	—
Fibrosis, lamina propria (Masson trichrome staining)	—	—	—	—	—	—	—	—	—	—	—	—	—	—	—	—	—	—	—	—	3	—	2	3	3	—	3	3	2	—
Mesentery (H&E and Masson trichrome staining)	—	—	—	—	—	—	—	—	—	—	—	—	—	—	—	—	—	—	—	—	—	—	—	—	—	—	—	—	—	—
Spleen																														
Decreased pigmentation (hemosiderin)	—	—	—	—	—	—	—	—	—	—	—	—	—	—	—	—	—	—	—	—	2	—	2	1	1	1	—	2	1	—
Liver																														
Proliferation, bile duct	—	—	—	—	—	—	—	—	—	—	—	—	—	—	—	—	—	—	—	—	2	1	1	1	1	1	2	3	3	1
Cell infiltration (lymphoid cell), periportal	—	—	—	—	—	—	—	—	—	—	1	1	1	—	—	—	—	—	—	—	2	1	2	1	2	1	1	3	2	1
Pigmentation (brown, granular), periportal	—	—	—	—	—	—	—	—	—	—	—	1	—	—	—	—	—	—	—	—	2	1	2	2	2	1	2	2	1	1
Pigmentation (brown, granular), hepatocyte	—	—	—	—	—	—	—	—	—	—	1	1	—	—	1	—	1	—	1	1	2	1	1	2	2	1	3	1	2	2
Atrophy, hepatocyte	—	—	—	—	—	—	—	—	—	—	—	—	—	—	—	—	—	—	—	—	2	2	1	2	2	1	2	2	1	1
Decreased vacuole, periportal hepatocyte	—	—	—	—	—	—	—	—	—	—	—	—	—	—	—	—	—	—	—	—	3	2	2	3	3	3	3	2	2	2
Necrosis, focal	—	—	—	—	—	—	—	—	—	—	—	—	1	—	—	—	—	—	—	—	—	—	—	1	—	—	—	—	1	—
Inflammation, perivascular (portal vein), focal	—	—	—	—	—	—	—	—	—	—	—	—	1	—	—	—	—	—	—	—	—	—	—	—	—	—	—	—	—	—
Kidney																														
Mineralization, corticomedullary junction	—	—	—	—	1	1	1	—	1	1	1	—	1	—	1	2	2	—	—	1	2	3	3	2	2	1	3	2	2	2
Pigmentation (brown, granular), proximal tubule	—	—	—	—	—	—	—	—	—	—	1	1	1	1	1	1	1	1	1	1	2	2	2	2	2	2	2	2	3	2
Cast	—	—	—	—	P	—	—	—	—	—	—	—	—	—	—	—	—	—	P	—	—	—	—	—	—	—	—	—	—	—
Hyperplasia, urothelial, pelvis, focal	—	—	—	—	—	—	—	—	—	—	—	—	—	—	—	—	—	—	1	—	—	—	—	—	—	—	—	—	—	—
Regeneration, tubular	—	1	—	—	—	—	—	—	—	—	—	—	—	—	—	—	—	—	—	—	—	—	—	—	—	—	—	—	—	

—: no remarkable changes; 1 : minimal; 2: mild; 3 : moderate; P : present. Rat ID numbers : 01–10 (control), 11–21 (1% GF), and 31–41 (2% GF).

**Table 2:** Individual histopathological findings in female rats during the 3-month recovery period.

Dose	Control										1% GF								2% GF				
Rat ID no.	51	52	53	54	55	56	57	58	59	60	72	73	74	75	76	77	78	79	82	85	86	88	90
Tissue observation																							
Ileum (H&E and Masson trichrome staining)	—	—	—	—	—	—	—	—	—	—	—	—	—	—	—	—	—	—	—	—	—	—	—
Colon																							
Deposit, eosinophilic, lamina propria	—	—	—	—	—	—	—	—	—	—	—	—	—	—	—	—	—	—	—	—	1	—	—
Fibrosis, mesocolon, perivenus region	—	—	—	—	—	—	—	—	—	—	—	—	—	—	—	—	—	—	—	—	1	—	—
Fibrosis, lamina propria (Masson trichrome staining)	—	—	—	—	—	—	—	—	—	—	—	—	—	—	—	—	—	—	—	—	1	—	—
Mesentery (H&E and Masson trichrome staining)	—	—	—	—	—	—	—	—	—	—	—	—	—	—	—	—	—	—	—	—	—	—	—
Spleen																							
Decreased pigmentation (hemosiderin)	—	—	—	—	—	—	—	—	—	—	—	—	—	—	—	—	—	—	—	2	—	1	—
Increased extramedullary hematopoiesis	—	—	—	—	—	—	—	—	—	—	—	—	—	—	—	—	—	—	1	—	—	—	—
Decreased lymphoid cell PALS	—	—	—	—	—	—	—	—	—	—	—	—	—	—	—	—	—	—	—	—	—	1	—
Liver																							
Cell infiltration (lymphoid cell), periportal	—	—	1	—	—	—	—	—	—	—	—	—	—	—	—	—	—	—	2	1	1	1	1
Pigmentation (brown, granular), hepatocyte	—	—	—	—	—	—	—	—	—	—	1	1	1	1	1	1	1	1	1	1	1	1	1
Atrophy, hepatocyte, centrilobular	—	—	—	—	—	—	—	—	—	—	—	—	—	—	—	—	—	—	1	1	2	1	1
Decreased vacuole, periportal hepatocyte	—	—	—	—	—	—	—	—	—	—	—	—	—	—	—	—	—	—	—	1	2	—	—
Vacuolation, hepatocellurar, focal	—	—	—	1	—	—	—	—	—	—	—	—	—	—	—	—	—	—	—	—	—	—	—
Vacuolation, hepatocellurar, periportal	—	—	—	—	1	—	—	—	1	—	1	—	—	1	1	—	—	—	1	1	—	—	—
Regeneration, hepatocellular, focal	—	—	—	—	—	—	—	—	—	—	1	1	1	1	1	1	1	1	1	1	1	1	1
Adrenal																							
Congestion/hemorrhage, cortex	—	—	—	—	—	—	—	—	—	—	—	—	—	—	—	—	—	—	—	—	—	1	—
Kidney																							
Mineralization, corticomedullary junction	2	1	—	—	1	—	—	1	1	1	2	2	1	1	1	2	1	1	2	2	1	2	2
Pigmentation (brown, granular), proximal tubule	—	—	—	—	—	—	—	—	—	—	1	1	1	1	1	1	1	1	2	1	2	1	2
Cast, focal	—	P	—	—	—	—	—	—	—	—	P	—	—	P	P	—	—	P	—	P	2	—	—
Fibrosis, cortex, focal	—	—	—	—	—	—	—	—	—	—	—	—	—	—	—	—	—	—	—	1	—	—	—
Chronic progressive nephritis	—	—	—	—	—	—	—	—	—	—	—	—	—	—	—	—	—	—	—	—	1	—	—

— : no remarkable changes; 1 : minimal; 2 : mild; P : present. Rat ID numbers : 51–60 (control), 72–79 (1% GF), and 82–90 (2% GF).

## Data Availability

The data used to support the findings of this study are included within the article.
